# Supplementary material for: Analysis of Host-Specific Differentiation of Puccinia striiformis in the South and North-West of the European Part of Russia
Source: Plants (Basel). 2021 Nov 18;10(11):2497. doi: 10.3390/plants10112497 (PMC8622514; doi:10.3390/plants10112497)
Supplement: Supplementary file 1 [file plants-10-02497-s001.zip › plants-1431683-supplementary.pdf]

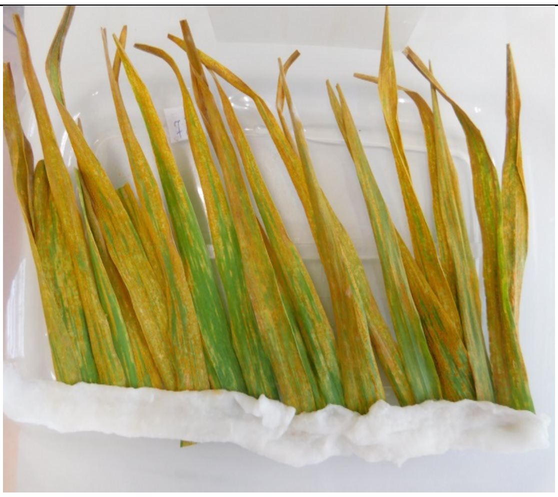

a

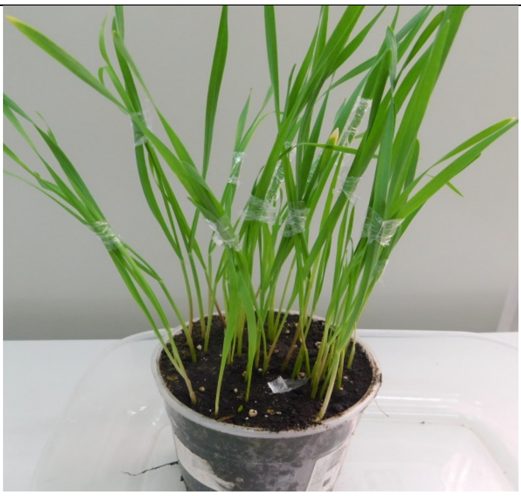

b

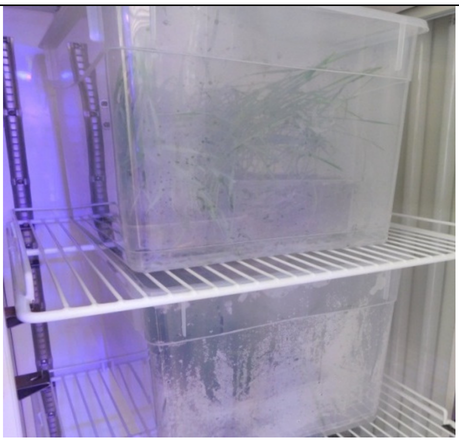

c

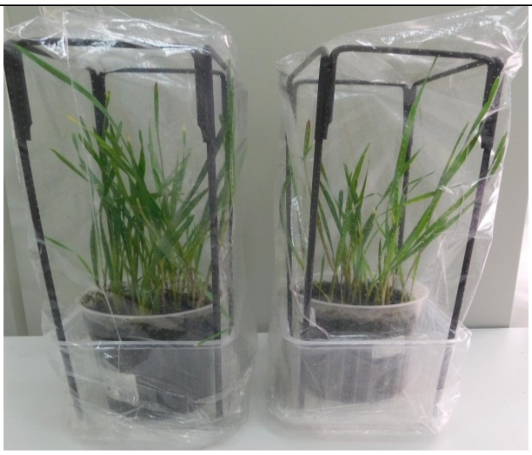

d

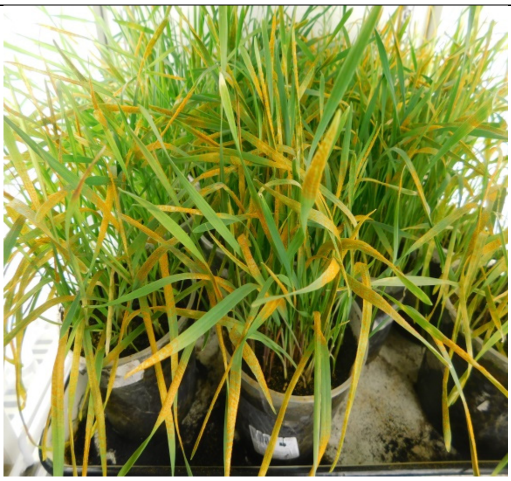

e

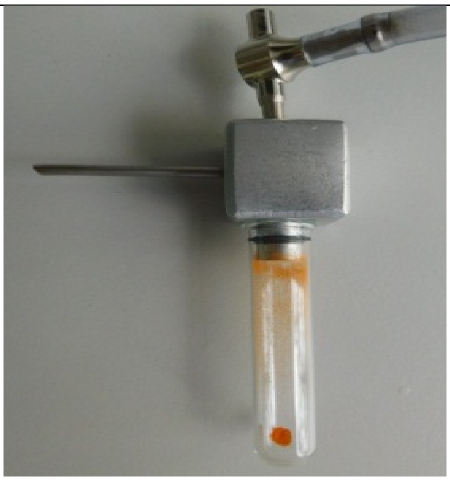

f

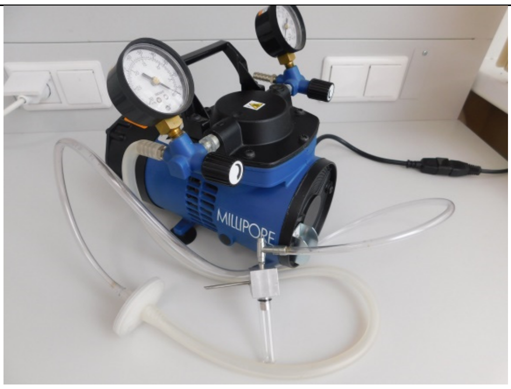

g

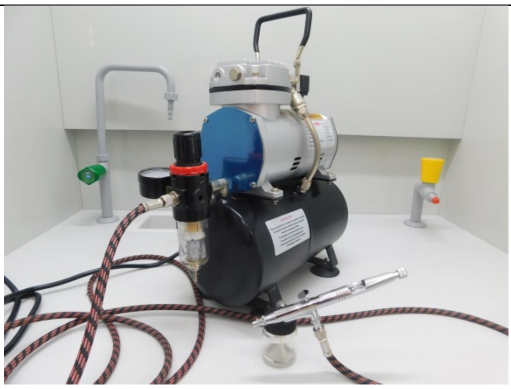

h

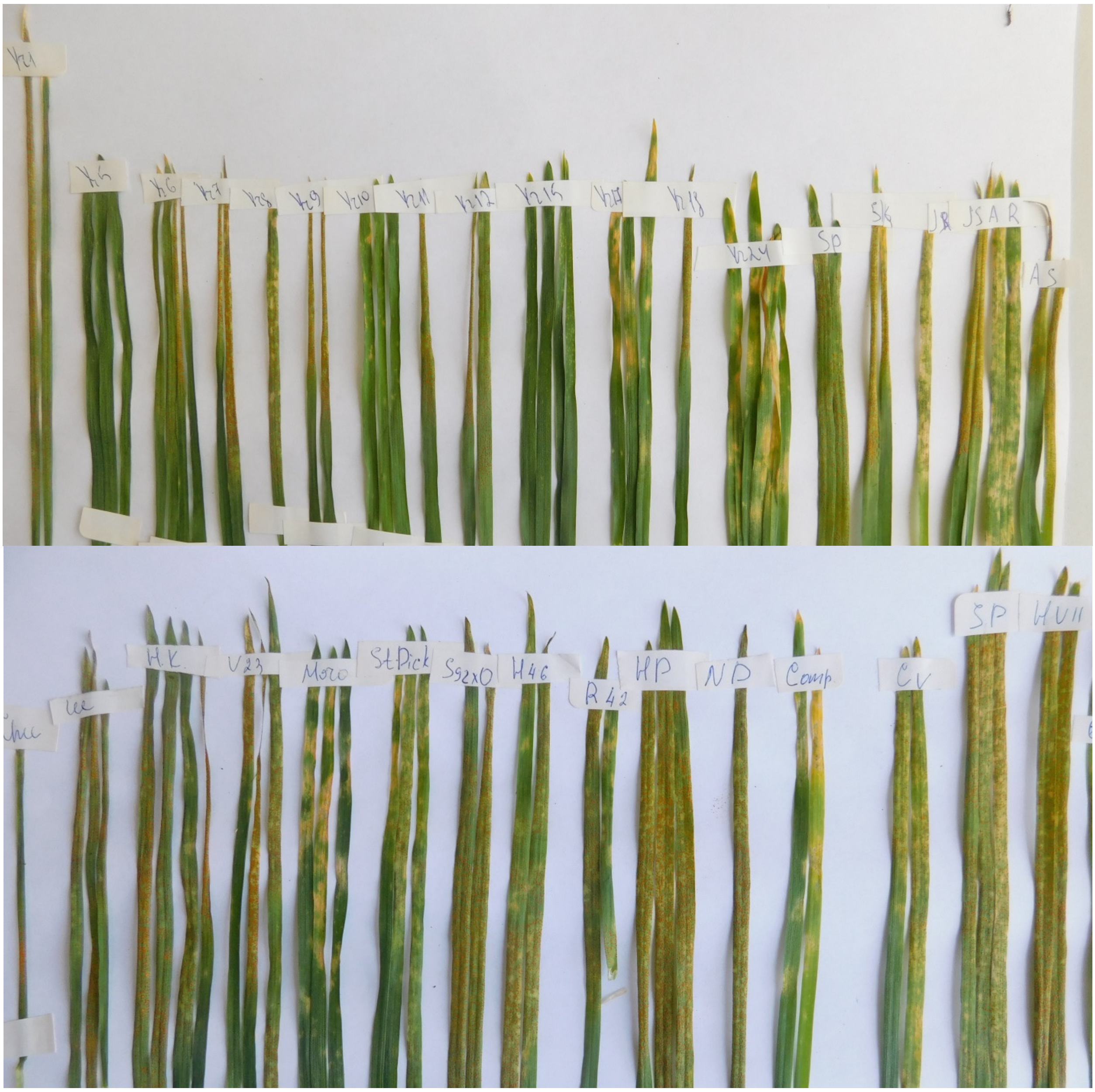

i

Figure S1. Laboratory studies of *Puccinia striiformis*
a) recovering urediniospores; b) multiplication of recovering urediniospores; c) inoculated plants in Versatile Environmental Test Chamber MLR-352H (SANYO ElectricCo., Ltd.); d) isolated plants, e) plants in 15–20 days after inoculation; f) harvesting urediniospores with a cyclone spore collector (g); h) airbrush spray gun for inoculation; i) virulence pattern on entire set of differentials.
